# A Crucial Role of Mitochondrial Dynamics in Dehydration Resistance in *Saccharomyces cerevisiae*

**DOI:** 10.3390/ijms22094607

**Published:** 2021-04-27

**Authors:** Chang-Lin Chen, Ying-Chieh Chen, Wei-Ling Huang, Steven Lin, Rimantas Daugelavičius, Alexander Rapoport, Chuang-Rung Chang

**Affiliations:** 1 Institute of Biotechnology, National Tsing Hua University, 101, Section 2, Kuang-Fu Road, Hsinchu City 300044, Taiwan; jasonh456@gmail.com (C.-L.C.); b09160187196418b@hotmail.com (Y.-C.C.); weiling850303@gmail.com (W.-L.H.); 2 Institute of Biological Chemistry, Academia Sinica, 128, Section 2, Academia Road, Nankang, Taipei 115201, Taiwan; stevenlin@gate.sinica.edu.tw; 3 Department of Biochemistry, Faculty of Natural Science, Vytautas Magnus University, Vileikos 8, 44404 Kaunas, Lithuania; rimantas.daugelavicius@vdu.lt; 4 Institute of Microbiology and Biotechnology, University of Latvia, Jelgavas Str., 1-537, LV-1004 Riga, Latvia; rapoport@mail.eunet.lv

**Keywords:** mitochondria, dehydration, dynamics, yeast

## Abstract

Mitochondria are dynamic organelles as they continuously undergo fission and fusion. These dynamic processes conduct not only mitochondrial network morphology but also activity regulation and quality control. *Saccharomyces cerevisiae* has a remarkable capacity to resist stress from dehydration/rehydration. Although mitochondria are noted for their role in desiccation tolerance, the mechanisms underlying these processes remains obscure. Here, we report that yeast cells that went through stationary growth phase have a better survival rate after dehydration/rehydration. Dynamic defective yeast cells with reduced mitochondrial genome cannot maintain the mitochondrial activity and survival rate of wild type cells. Our results demonstrate that yeast cells balance mitochondrial fusion and fission according to growth conditions, and the ability to adjust dynamic behavior aids the dehydration resistance by preserving mitochondria.

## 1. Introduction

*Saccharomyces cerevisiae* is an anhydrobiotic fungus that can survive under a long-term desiccated condition. Yeast cells can resist mechanical, oxidative, osmotic, or other stressful constraints under a desiccated condition [1,2]. Since preserving yeast cells in a dry condition is critical for winery, food industry, and medical applications [3], it is valuable for us to understand the underlying mechanisms of how yeast cells overcome a desiccation condition.

Trehalose, sugar alcohol and hydrophilin (Late embryogenesis abundant protein (LEA) in plants) can be used to substitute water molecules to maintain structure and activity of cell components like lipid bilayer, proteins, and nucleic acids in yeast cells during dehydration [4,5,6,7,8,9,10]. Additionally, cellular organelle maintenance and inheritance are involved in desiccation [11]. It is known that during dehydration and rehydration, cells with a better capacity to preserve functional organelles and membranes have a better chance to survive [12].

Mitochondria are essential organelles responsible for energy production, and are also involved in calcium homeostasis, fatty acid oxidation, lipid synthesis, and many other critical cellular metabolic reactions [13,14,15,16]. Previous studies have indicated that mitochondria integrity is closely correlated to desiccation tolerance [17,18,19]. Mitochondria are dynamic organelles [20]. These organelles are not static; instead, they continue to go through fusion, fission, and transport [21]. The dynamic processes are important for mitochondria quality control [22]. Damaged mitochondria are eliminated through sophistically balancing between these dynamic processes. In yeast cells, *DNM1* encodes a dynamin-related GTPase that performs mitochondrial fission [23]. Dnm1 is recruited by Fis1, a Dnm1 receptor, to mitochondrial tubules and assembles as ring structure to constrict and cleave both outer and inner membrane of mitochondria. On the other hand, *FZO1* encodes a transmembrane GTPase on the mitochondrial outer membrane for collaborating with Mgm1 and Ugo1 to fuse outer and inner mitochondrial membranes [24]. Several signaling pathways have been implicated in the regulation of mitochondrial dynamics, such as post-translational modifications of conventional fusion/fission factor and ER/mitochondria contacts [25,26,27].

Although mitochondrial integrity is critical for desiccation tolerance, how cells maintain mitochondria during dehydration/rehydration remains unclear. To address this issue, we focused on the contributions of dynamic factors in different growth conditions for dehydration resistance. Our results demonstrated that disrupting mitochondrial dynamics in stationary phase severely hampered the capability of cells to deal with dehydration/rehydration stress. A well-maintained dynamic balance of fusion and fission supports mitochondrial genome integrity and functions that help in dehydration resistance.

## 2. Results

### 2.1. Stationary Growth Aids Resistance to Dehydration

**Figure 1:**
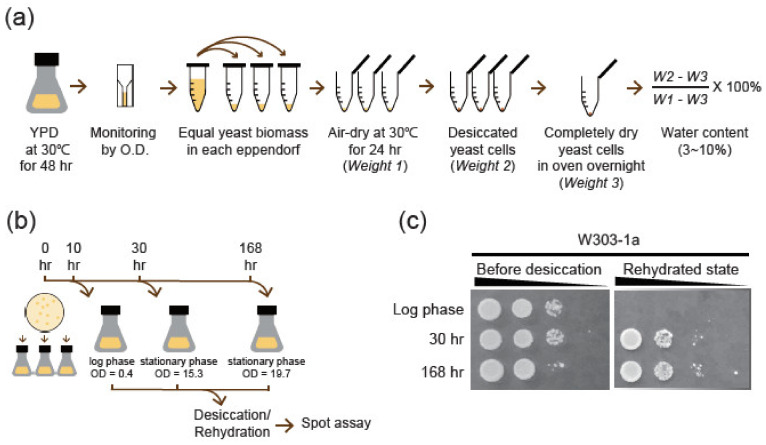
Yeast cells are more resistant to dehydration after stationary growth phase. (**a**) Dehydration procedure for yeast cells and the calculation of remaining water of the dehydrated cells. (**b**) Yeast cell samples were taken from log and stationary growth phases as depicted for dehydration/rehydration. (**c**) Resistance to dehydration is assayed by viability spotting assay.

We followed the dehydration and rehydration protocol to create anhydrobiosis state of cells as shown in Figure 1a to examine the desiccation tolerance of yeast cells [28]. Anhydrobiosis is a unique dormant state of cells under a highly dehydrated condition. The desiccated cells contained less than 10% water compared to cells before dehydration. To elucidate whether growth condition has an impact on desiccation tolerance, exponential and stationary phase cell samples were collected and dehydrated as shown in Figure 1a,b. We used spot assay with serially diluted cells to compare the desiccation survival rate of different samples after rehydration. Whether cells enter diauxic shift or not, samples taken from stationary growth phase have a much better survival rate after rehydration than those without (Figure 1c). These results demonstrated that pre-dehydration growth condition would affect survival rate and desiccation tolerance.

### 2.2. Mitochondrial Network Exhibited Fragmented Morphology in Stationary Phase

**Figure 2:**
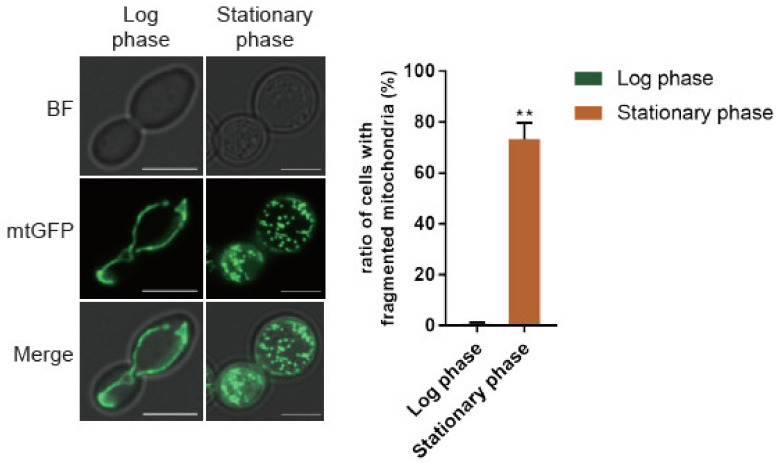
The balance of mitochondrial dynamics shifts toward fission in stationary phase. Representative mitochondrial network morphology in both log and stationary growth phases. Three independent trials of cells were collected, and the difference of the percentage of cells containing fragmented mitochondria were assayed by paired student *t* test. **: *p* value <0.01.

To clarify whether mitochondrial dynamics contribute to dehydration resistance for cells that went through stationary growth, we targeted GFP to mitochondria for examining the organellar network morphology. Mitochondrial network morphology is determined by the dynamic balance. Sample cells were taken from log and stationary growth phases. We found that the majority of cells in log phase contain the tubular form of mitochondrial network. Approximately 73% of cells on average from the stationary phase were found to have fragmented mitochondria (Figure 2). The results led us to speculate a regulatory mechanism to tune the balance of mitochondrial dynamics during stationary phase growth. Dynamic fusion and fission are known to maintain active mitochondria [29]. This finding led us to suspect that being able to adjust the dynamic balance may contribute to maintenance of mitochondria under stress of dehydration/rehydration processes.

### 2.3. Disrupted Mitochondrial Dynamics Impairs Resistance to Dehydration

**Figure 3:**
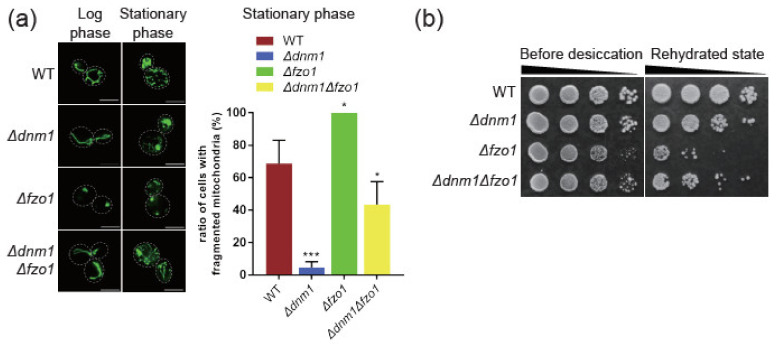
Mitochondrial dynamics defects attenuate dehydration resistance. Deletion of mitochondrial fusion Fzo1 and fission Dnm1 factors reduce cell survival after dehydration/rehydration. (**a**) Representative mitochondria network morphology of wild type and dynamic defect strains from samples taken from the stationary phase. Quantitative results are from three individual trials of experiments; at least 100 cells were examined in each trial. One-way ANOVA was used for statistical analysis. (**b**) Spot assay results for cell viability before and after dehydration. A ten-foldserial dilution of cells were spot on the YPD plates. *: *p* value <0.1; ***: *p* value <0.001.

Mitochondria fission in yeast cells is mediated by Dnm1. On the other hand, fusion is by Fzo1. We constructed dynamic defect strains *Δdnm1, Δfzo1* and *Δdnm1 Δfzo1* double deletion strains to elucidate the roles of mitochondrial dynamics in the dehydration resistance during the stationary growth phase. *Δdnm1* cells contained a hyperfused mitochondrial network, while *Δfzo1* cells harbored fragmented mitochondria. *Δdnm1 Δfzo1* cells possess the tubular form of mitochondria (Figure 3a). These results confirmed that deletion of conventional fusion/fission factors did disrupt mitochondrial dynamics. A noticeable result is that double deletion strain *Δdnm1 Δfzo1* possessed similar mitochondria as the wild type cells. To examine whether the change of dynamic balance in the stationary phase contributed to desiccation tolerance, we examined the survival rate of *Δdnm1, Δfzo1* and *Δdnm1 Δfzo1* cells that went through stationary phase growth and dehydration/rehydration processes. Based on the semi-quantitative colony numbers of the serial dilution spotting assay, we found that the *Δdnm1* strain had about ten folds fewer cells after dehydration compared to the wild type strain. *Δfzo1* strain had the worst survival rate among mutant strains. Surprisingly, the double deletion strain *Δdnm1 Δfzo1* had a similar survival rate as the wild type cells (Figure 3b and Supplemental Figure S1). The cell survival results after dehydration/rehydration supported the hypothesis that mitochondrial dynamics are critical for desiccation tolerance.

### 2.4. Dynamic Processes Mediated mtDNA Maintenance Is Correlated with Dehydration Resistance

**Figure 4:**
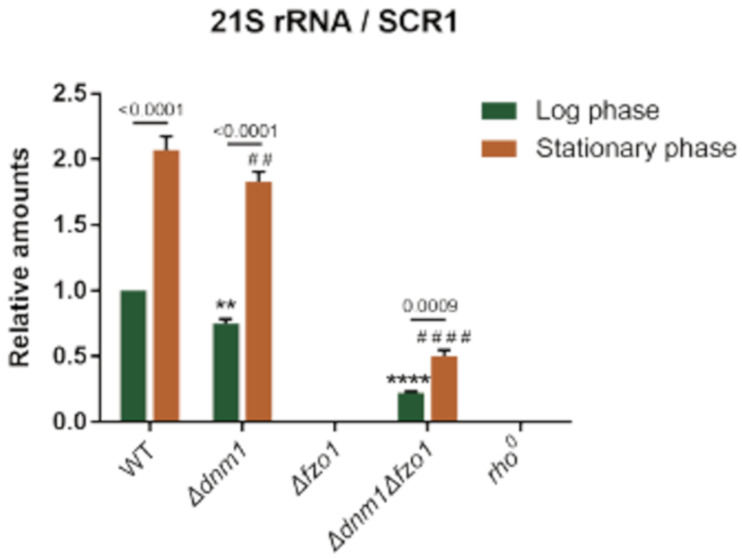
Dehydration resistance is proportion to mtDNA copy number. Quantitative PCR was used to examine the ratio of mitochondrial (21S rRNA) and nuclear genome (*SCR1*) copy number. All results were normalized to log growth phase wild type strain. The two-way ANOVA multiple comparison was used for statistics analysis. **/^##^: *p* value <0.01; ****/^####^: *p* value <0.0001.

Alterations of mitochondrial dynamics resulting in a loss of mtDNA and/or aberrancies in mt-nucleoid morphology have been reported in both mammalian and yeast cells [30,31,32]. To characterize the role of mtDNA maintenance in desiccation tolerance, we examined the mtDNA copy numbers of wild type, *Δdnm1, Δfzo1* and *Δdnm1 Δfzo1* strains in both log phase and stationary phase cells. Comparing the quantitative PCR results of mtDNA, we found *Δdnm1, Δfzo1* and *Δdnm1 Δfzo1* strains possessing less mtDNA than wild type cells in both log and stationary growth conditions (Figure 4). Especially, mtDNA copy number was severely reduced in the *Δfzo1* strain. In addition, cell samples taken from stationary growth phase have at least 2 folds higher mtDNA copy number than those from the log phase. These results indicated that maintaining the ability of mitochondria to fuse and divide helps to maintain mitochondrial genome. Stationary phase growth was apparently linked to higher mtDNA copy number. Higher mtDNA copy number equated with better survival rate after dehydration/rehydration as shown in Figure 3b.

### 2.5. Disrupted Dynamic Processes Change Mitochondrial Activity

**Figure 5:**
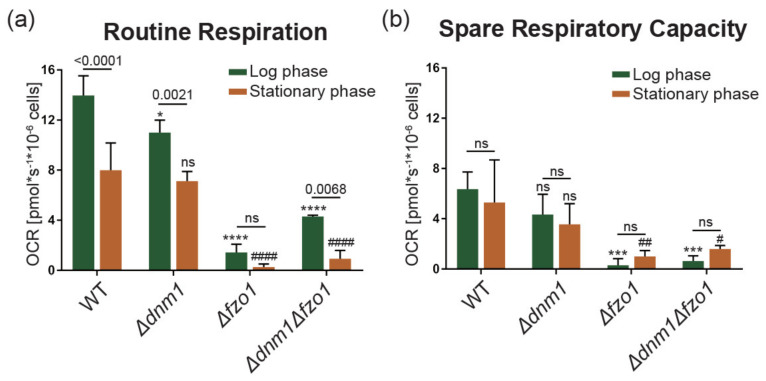
Mitochondrial respiration in wild type and mutant cells. The oxygen consumption rate of different yeast strains was measured in both log (green) and stationary growth phases (orange). Two-way ANOVA multiple comparison was used for statistics analysis. The individual growth phase comparison was calculated based on mutant strain to wild type strain and marked with either * or #. The *p* value of individual strain for different growth phase was depicted in the figure. (**a**) Routine respiration of different strains. (**b**) Spare respiratory capacity based on the results of coupling control protocol. */^#^: *p* value <0.1; ^##^: *p* value <0.01; ***: *p* value <0.001; ****/^####^: *p* value <0.0001.

The mitochondria genome encodes critical components for respiration complexes. To clarify the mitochondria activity of wild type and dynamic defect cells in both log and stationary growth phases, we applied the Oroboros Oxygraph 2K^®^ high-resolution respirometer to record the oxygen consumption rate. Except for monitoring the routine respiration, a coupling control protocol (CCP) was used to challenge different oxidative phosphorylation complexes for evaluating respiration capacity. Coupling control protocol added oxidative phosphorylation inhibitors, triethyltin bromide (TET), Carbonyl cyanide-4-(trifluoromethoxy)phenylhydrazone (FCCP) and antimycin (AntA) sequentially while assaying oxygen consumption rate [33,34]. We found that cells at stationary phase growth have lower routine respiration (Figure 5a). Strains with mitochondrial dynamics factor deletion have even lower routine respiration rate than normal. Despite the effect on routine respiration, the spare respiration capacity did not exhibit significant difference between log and stationary phase cells (Figure 5b).

## 3. Discussion

**Figure 6:**
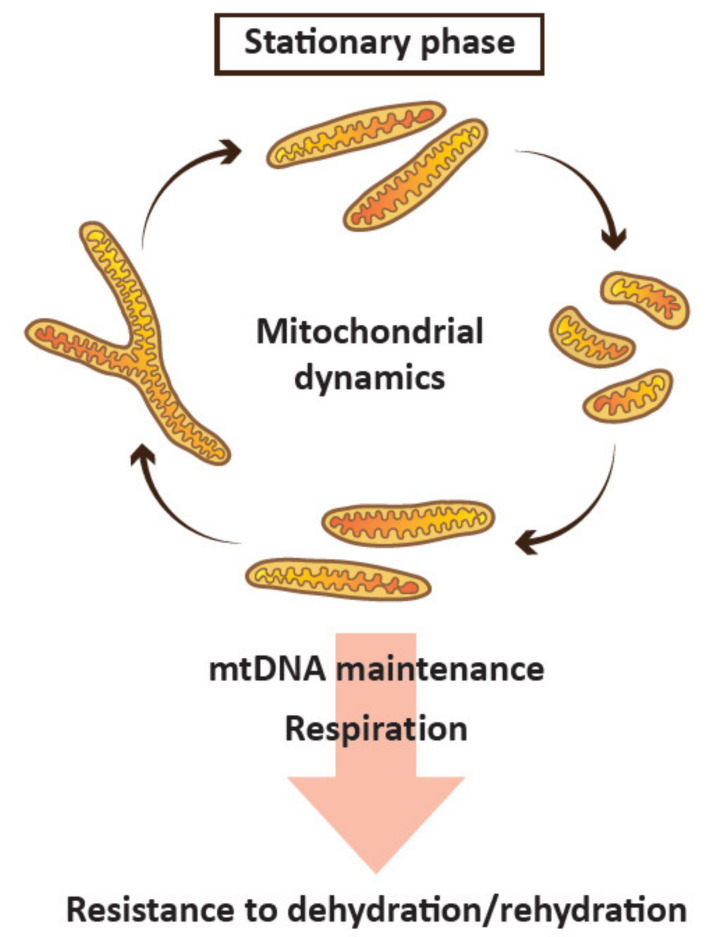
Dynamic fusion and fission aid dehydration resistance by preserving mitochondrial genome in stationary growth phase. Schematic showing that fusion and fission in stationary phase facilitate to preserve mitochondria, especially by maintaining mtDNA. The dynamic processes facilitate the dehydration resistance of yeast cells.

Yeast cells have a remarkable ability to survive the loss of almost all water and recover from dehydrated status. Previous studies have emphasized the importance of stationary phase in the resistance to dehydration [35]. Our results demonstrated one step further that maintaining the ability to adjust mitochondrial dynamics in stationary growth phase is critical for desiccation tolerance. Although we have not characterized what make wild type cells more resistant to dehydration, our results demonstrate that defects in adjusting mitochondrial dynamics caused a significantly lower survival rate after dehydration/rehydration. Dynamic fusion and fission defects caused a loss of mitochondrial genome and activity. Our results suggest that utilizing dynamic processes in stationary phase, which helps preserving mitochondrial genome and maintaining respiration activity, is critical for a cell’s resistance to dehydration (Figure 6 and Supplemental Figure S2).

### 3.1. Mitochondrial Dynamics Changes along with Growth and Nutrition Supply Conditions

Wild type yeast cells typically contain tubular mitochondria in exponential growth phase. Our results demonstrated mitochondrial network turned into fragmented during the stationary growth phase. The change in the balance of mitochondrial dynamics may be due to the alteration of nutrition condition in the medium. We suspect that the critical component is dextrose because yeast cells adjusting their metabolism depend on a carbon source [36]. Mechanisms that regulate mitochondrial morphogenesis and mitochondrial functions are varied under different specific extrinsic or intrinsic metabolic cues [16]. Our results showed that mitochondrial dynamics in stationary growth phase is beneficial for desiccation tolerance in yeast, and several reports also suggest fragmented mitochondria are more stress-resistant [37,38]. However, the signaling pathways responsible for the change of mitochondrial dynamics in the stationary phase remain to be elucidated.

### 3.2. Disrupted Fusion and Fission Balance Is Associated with mtDNA Loss and Lower Desiccation Tolerance

Being able to adjust mitochondrial dynamics is critical for cells while encountering environmental stress. In WT cells, fragmented mitochondria phenotype in stationary phase correlates with dehydration resistance. In contrast, the presence of more fragmented mitochondria in *Δfzo1* cells correlates with less resistance to dehydration (Figure 3). This result demonstrated that a well-maintained mitochondrial dynamics machinery contributes to dehydration resistance. Among the factors that influence mitochondrial dynamics, mtDNA copy number appears to be a potential indicator of resistance to dehydration, as more mtDNA in stationary phase and in wild type cells compared to *Δdnm1, Δfzo1* and *Δdnm1 Δfzo1* mutants leads to better resistance to dehydration. We suspect that cells with a higher mtDNA copy number have a larger reservoir of mitochondria genome to consume while encountering harsh dehydration/rehydration environmental stress (Figure 4 and Figure 5). The results of double deletion strain*Δdnm1 Δfzo1* with slightly higher mtDNA copy number along with a higher survival rate after dehydration/rehydration supported our speculation about mtDNA copy number in dehydration resistance. The mitochondrial network morphology of *Δdnm1 Δfzo1* cells in a stationary phase was not like *Δdnm1* or *Δfzo1* single deletion strains (Figure 3). The *Δdnm1 Δfzo1* double deletion strains morphology in a stationary phase is like that in wild type cells. This result not only implied that a minor fusion/fission machinery exists, but that dynamic process per se is critical for resistance to dehydration. The uncharacterized fusion/fission machineries revealed their function only when the conventional fusion/fission was completely blocked.

### 3.3. Desiccation Tolerance Relies on Well-Regulated Mitochondrial Dynamics to Maintain Organellar Integrity and Signaling

Researches have shown that mitochondrial fission facilitates mitochondrial inheritance [39] and elimination of dysfunctional mitochondria [40,41]. Studies from Osman et al. indicated that fusion and fission defects cause loss of mtDNA integrity with structural variations [42]. Our results support this notion. Deletion of dynamics factors caused reduced mtDNA copy number and respiration capacity in both log and stationary growth phases (Figure 5). The attenuated mitochondria eventually caused compromised dehydration resistance. A notable result is that mtDNA copy number is generally higher in stationary cells than log phase cells. We suspect this may be related to the compensation of mitochondria under stationary stress. Thus, additional factors involved in maintaining mitochondria integrity and dehydration resistance remain to be characterized.

In summary, we found that mitochondrial dynamics machinery contributes to desiccation tolerance in yeast cells by maintaining mitochondria integrity. Preservation of yeast strains, plant seeds and other organisms frequently involves dehydration of cells. Our results suggest that better survival rate after dehydration/rehydration processes requires a better approach to maintain the organelle’s integrity.

## 4. Materials and Methods

### 4.1. Yeast Strain Constructions and Growth Conditions

We used *Saccharomyces cerevisiae* W303-1a as the parental wild type strain in this study to construct deletion strains. All the strains used in this study are listed in Supplemental Table S1. W303-1a isogenic deletion mutants were generated by direct PCR replacement with either auxotrophic (histidine, *HIS3MX6*) or antibiotic (hygromycin B phosphotransferase, *HPHMX6*) cassettes. Mitochondrial genome depletion strain *rho^0^* were generated by ethidium bromide treatment [43] and selected by non-fermentable carbon source medium. Liquid YPD (1% Yeast extract, 2% Bacto-Peptone, 2% Dextrose) and synthetic complete medium were prepared for yeast cultures at 30 °C in this study.

### 4.2. Desiccation and Rehydration

Desiccation protocol is as Figure 1 [28]. In short, fresh yeast colonies were picked from YPD agar plate (2% agarose) and culture in 200 mL liquid YPD at 30 °C, 220 rpm. Freshy yeast inoculum took about 44–48 h to grow to stationary phase. Yeast cells were then centrifuged and washed with PBS (Phosphate Buffered Saline 1×; pH 7.0). A small volume of PBS (1×; pH 7.0; about 15–20 mL) was added to resuspend yeast cells. Equal biomass in individual Eppendorf was aliquoted based on spectrophotometry (O.D. 600). Cells were centrifuged again to discard supernatant. Pellets were dried in incubator at 30 °C for 24 h or till pellets could be easily moved. Water content of pellets was measured by calculating remnant water weight in microfuge tubes. Based on the weighting, each patch of dehydrated yeast cells contained 6–10% water content at this state compared to those cells without incubation. Rehydration solution was either YPD or 1× PBS depended on the experiments described.

### 4.3. Cell Viability Assay

*Spot Assay:* Yeast cells were cultured and dehydrated as described previously. Samples were taken as shown in Figure 1 and serial ten-fold dilutions were done in a 96-well plate. All dilutions were spotted by multi-blot replicator VP407AH on YPD agar plate incubating at 30 °C for 2 days before taking images.

*Methylene Blue Staining:* Yeast cells were cultured and dehydrated as described previously. Dry yeasts were rehydrated with YPD for 20 min to revive cells. These rehydrated cells were centrifuged and resuspended with methylene blue (0.1 mg·mL^−1^ stock solution, dissolved in PBS solution) for 15 min. Cell viability ratio was measured by counting above 500 cells in each strain [44].

### 4.4. Microscopy

The Axioskop 2 mot plus fluorescence microscope (Carl Zeiss, Oberkochen, Germany) along with camera system was under Zeiss Zen software control. Live cells samples were cultured to experimental phase for imaging. To visualize mitochondria under fluorescent microscope, we transformed two micron-based plasmid pVT100U-mtGFP to overexpress GFP fused with Su9(1–69) targeting to mitochondrial matrix [45]. Images were analyzed by either Axiovision 4.6 or Zen 2012 version.

### 4.5. Quantitative PCR for mtDNA

DNA extraction was carried out by traditional phenol-chloroform approach. Quantitative PCR (qPCR) was conducted using SensiFAST™ SYBR^®^ Hi-ROX Kit (Bioline) followed by protocol provided by Bioline (https://www.bioline.com/sensifast-sybr-hi-rox-kit.html (accessed on 26 April 2021)). The amplified sequences are 21S rRNA on mtDNA and SCR1 on chromosome V in yeast nuclear genome. We quantify the amount of mtDNA copy number per nuclear genome with following primers: mtDNA forward primer: 5′-CCGTAATGTAGACCGACTCAG-3′, mtDNA reverse primer: 5′-TGGAGCAGAGTTCACACCTTA-3′, nuclear DNA forward primer: 5′-CGCGGCTAGACACGGATT-3′, nuclear DNA reverse primer: 5′-GCACGGTGCGGAATAGAGAA-3′. Data of qPCR were analyzed by comparing threshold cycles with ΔΔC(t) by StepOnePlus Detection System (Applied Biosystems, Foster, CA, USA).

### 4.6. High Resolution Respirometry

Yeast mitochondrial oxygen consumption was measured at 30 °C by high resolution Oxygraph-2k (OROBOROS Instruments, Innsbruck, Austria). We used culture medium as assay buffer and respiration complexes inhibitors based on coupling control protocol to measure mitochondrial respiration [33]. Yeast cells were placed in a 2 mL chamber at a final concentration of 5 × 10^6^ cells/mL. After basal respiration was recorded, Triethyltin (TET, 150 µM), Carbonyl cyanide p-trifluoromethoxy phenylhydrazone (FCCP, 3, 5, 7 µM), and Antimycin A (2 µM) were used for measuring respiration at leak state, proton gradient uncoupled state, and electron transport chain blocking state, respectively. Inhibitors injection protocol was modified by the previous study [34]. Oxygen consumption rate, OCR, (in pmol/s × 10^6^ cells) is directly proportional to oxygen consumption, which is calculated by the decrease slope of oxygen concentration. Data were continuously recorded and analyzed by DatLab 6 software.

### 4.7. Statistical Analysis

All experimental results shown here are the results of three biological replicates. To classify fragmented mitochondria, >200 cells were examined in each trail. Statistical analysis was performed using GraphPad Prism 7 (GraphPad Software, California USA). Histograms present the mean; error bars represent the standard deviation. Specified methods were used to calculate *p* value as mentioned in the figure legends. Statistical significance is determined by *p* < 0.05. *p* value: <0.1 (* or ^#^), <0.01 (** or ^##^), <0.001 (*** or ^###^), <0.0001 (**** or ^####^).

## Data Availability

The data that support the findings of this study are available from the corresponding author upon reasonable request.

## Supplementary Materials

The following are available online at https://www.mdpi.com/article/10.3390/ijms22094607/s1.
