# Supplementary material for: A Crucial Role of Mitochondrial Dynamics in Dehydration Resistance in Saccharomyces cerevisiae
Source: Int J Mol Sci. 2021 Apr 27;22(9):4607. doi: 10.3390/ijms22094607 (PMC8124315; doi:10.3390/ijms22094607)
Supplement: Supplementary file 1 [file ijms-22-04607-s001.zip › ijms-1144016-supplementary.pdf]

Supplemental Table 1. Strain list in this study

Name	Genotype
W303-1a	*MATa, leu2 trp1 can1 ura3 ade2 his3*
CYC027	W303-1a, *dnm1::HIS3*
CYC024	W303-1a, *fzo1::HPH*
CYC087	W303-1a, *dnm1::HIS3, fzo1::HPH*
CL022	W303-1a, *rho^0*

Supplemental Figure 1.

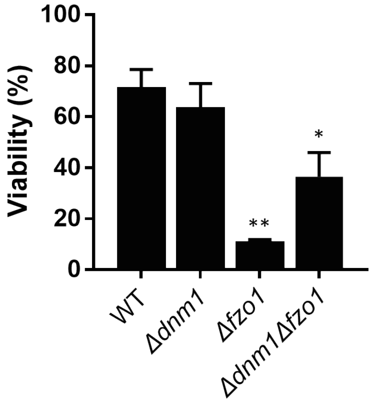

Figure S1. *Resistance to dehydration by yeast viability*

Wild type and mitochondrial dynamics deficient strains *Δdnm1, Δfzo1*, *Δdnm1 Δfzo1* were assayed by methylene blue staining. For each strain in a trial, more than 500 cells were counted. **, $P = 0.0013$; *, $P = 0.0291$ by one-way ANOVA.

Supplemental Figure 2.

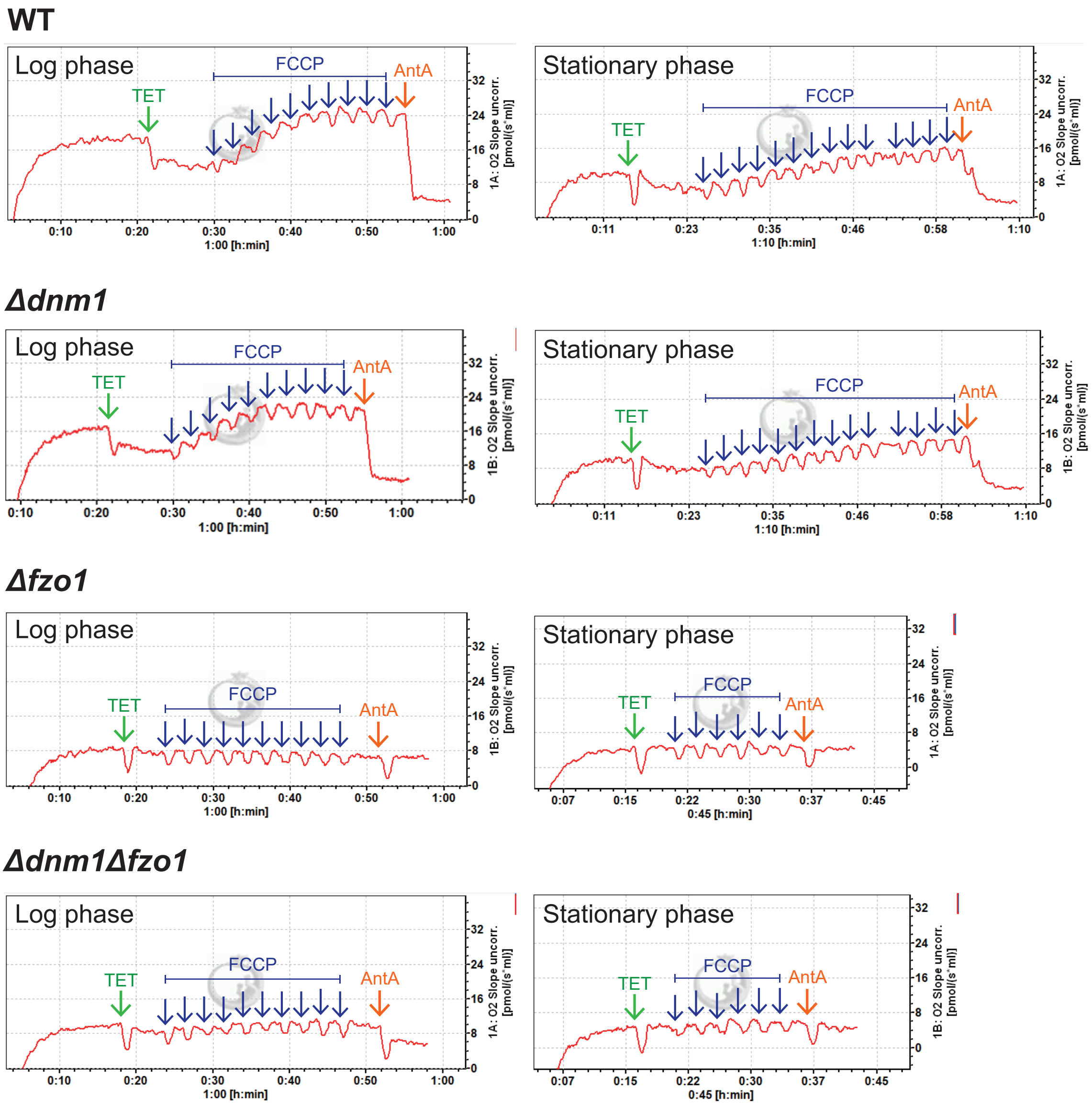

Figure S2. Representative yeast mitochondrial oxygen consumption rate by Oroboros Oxygraph-2k.

Wild type and mitochondrial dynamics deficient strains *Δdnm1, Δfzo1*, *Δdnm1 Δfzo1* were assayed in log phase and stationary phase. For each trial, we used YPD as assay buffer with $5*10^6$ cells per ml. Inhibitors were injected once the routine OCR value is stable. FCCP was injected by titration. Arrows indicate the time points of inhibitors injection. TET, triethyltin; FCCP, Carbonyl cyanide p-trifluoromethoxy phenylhydrazone; AntA, Antimycin A.
